# Evidence-Based Approaches in Clinical Orthodontics: A Narrative Review

**DOI:** 10.7759/cureus.91408

**Published:** 2025-09-01

**Authors:** Preethy S Nair, Parvathy Ghosh, Sapna Varma N.K., Ajith Vallikat Velath

**Affiliations:** 1 Orthodontics and Dentofacial Orthopedics, Amrita School of Dentistry, Amrita Institute of Medical Sciences, Kochi, IND

**Keywords:** clear aligners, evidence, evidence pyramid, extractions, facemask, functional appliance, malocclusion, maxillary expansion, rapid maxillary expansion (rme), tmj

## Abstract

Evidence-based practice in clinical orthodontics combines scientific research, clinical experience, and patient values to enhance treatment outcomes. This narrative review is based on evidence primarily from systematic reviews and randomized controlled trials, supplemented by relevant high-quality studies. Articles were selected to emphasize higher levels of clinical evidence in orthodontic practice, highlighting evidence-supported approaches in orthodontic care. Functional appliances such as Twin Block and MARA are effective for Class II malocclusion, promoting skeletal changes during growth and improving airway dimensions. Early treatment of Class III malocclusions using rapid maxillary expansion with facemasks, especially with skeletal anchorage, improves maxillary advancement. Orthognathic surgery, including the surgery-first technique and digital planning, efficiently addresses severe discrepancies. Temporary anchorage devices improve anchorage and allow for precise, complex movements using three-dimensional guidance. Airway improvements are noted following orthodontic and surgical interventions in children. Orthodontic treatment shows a neutral effect on temporomandibular disorders, though functional appliances may help. Clear aligners provide aesthetic and periodontal benefits but have limitations in complex cases. The future of orthodontics depends on the integration of evidence-based decision-making, developing technology, and interdisciplinary collaboration.

## Introduction and background

Recently, the healthcare industry, including dentistry, has increasingly embraced evidence-based practice to improve patient care and encourage practitioners to integrate the best available scientific evidence, clinical expertise, and patient preferences into their decision-making processes. Adopting evidence-based orthodontic practice is challenging, as it requires practitioners to stay updated with evolving research and prioritize high-level evidence such as randomized clinical trials and systematic reviews. Balancing evidence with factors such as availability, affordability, and acceptability is critical for providing the best possible patient care [1].

This review aims to evaluate current evidence on the effectiveness and limitations of different orthodontic interventions from the perspective of evidence-based practice in clinical orthodontics. This review also aims to analyze gaps and limitations in the existing literature and guide future research directions.

## Review

Methodology

Search Strategy

For this narrative review, a comprehensive literature search was performed to gather relevant publications addressing evidence-based practices in clinical orthodontics. The search was conducted across major electronic databases, including PubMed/MEDLINE, Scopus, and Google Scholar. To strengthen methodological quality, the search was refined to include systematic reviews as a key filter, while also identifying meta-analyses, randomized controlled trials, cohort and cross-sectional studies, and narrative reviews.

The strategy included combinations such as (“Orthodontics” OR “Orthodontic treatment” OR “Malocclusion” OR “Functional appliances” OR “Orthodontic Appliances, Functional” OR “Orthodontic Appliances, Fixed” AND (“Evidence-Based Dentistry” OR “Evidence-Based Practice” AND (“Clear aligners” OR “Orthodontic Appliances, Removable” OR “Functional appliance” OR “Rapid maxillary expansion” OR “Palatal Expansion Technique” OR “Orthognathic surgery” OR “Osteotomy, Sagittal Split Ramus” OR “Osteotomy, Le Fort” OR “Temporary anchorage devices” OR “Orthodontic Anchorage Procedures” OR “Retention” OR “Orthodontic Retainers” OR “Airway” OR “Airway Resistance” OR “Temporomandibular disorders”).

Inclusion Criteria

Articles written in the English language and published between 2000 and 2025 were included. Eligible study designs comprised systematic reviews and meta-analyses, randomized controlled trials, cohort studies, and narrative reviews. Priority was given to research on clear aligners, functional and fixed appliances, expansion methods, temporary anchorage devices (TADs), orthognathic surgery, and retention protocols, with outcomes such as occlusal correction, airway changes, temporomandibular disorders (TMD), periodontal or microbiome effects, aesthetics, stability, and appliance properties.

Exclusion Criteria

Articles were excluded if they were not published in the English language, not peer-reviewed, or outside the defined publication period (before 2000). Case reports, expert opinions, and editorials without original data, as well as studies unrelated to orthodontics or lacking systematic methodology, were also excluded.

Managing Class II malocclusion

Researchers estimate that the global prevalence of Class II malocclusion among individuals aged 0-19 years ranges between 19.56% and 20.2% [2]. In the Indian population, studies focusing on adolescents aged 13-17 years report varying prevalence rates, from 8.37% in Punjab to 17.6% in Kerala, likely reflecting regional and ethnic differences [3]. Treatment for Class II malocclusion typically involves restricting maxillary forward growth, stimulating mandibular growth, or combining both [1].

*History of Removable Functional Appliance*s

The evolution of functional appliances began in the early 20th century with Pierre Robin, who introduced the monobloc in 1902 to treat mandibular deficiencies. Around the same time, Alfred P. Rogers emphasized the role of the orofacial system, laying the groundwork for myofunctional therapy. In 1919, Andresen developed the activator appliance after observing his daughter’s spontaneous correction of Class II malocclusion. Later innovations included Rolf Fränkel’s tissue-borne appliance in the 1950s, Schwarz’s double-plate design in 1956, combining activator and expansion principles, and, finally, Clark’s Twin Block in 1982, which gained widespread acceptance for its clinical simplicity and effectiveness in sagittal correction [4].

*Effects of Removable Functional Appliance*s

Systematic reviews of randomized controlled trials involving growing individuals aged 6-18 years have demonstrated that removable functional appliances, such as Sander Bite Jumping, Twin Block, Bionator, Harvold Activator, and Frankel, are effective in increasing mandibular length, with Sander Bite Jumping showing the most pronounced effect. Supporting this, a clinical study in adolescents reported that Twin Block therapy led to significant skeletal, dentoalveolar, and soft tissue improvements in Class II, Division 1 malocclusion, particularly when treatment was initiated during CS-3 and CS-4 stages. These findings reinforce the effectiveness of Twin Block therapy during peak growth [5,6].

Effects of Removable Functional Appliances on the Airway

Multiple systematic reviews have demonstrated that functional appliances such as Twin Block, Monoblock, Bionator, and Andresen significantly increase upper and middle pharyngeal airway dimensions in growing patients with skeletal Class II malocclusion. Mandibular advancement and forward repositioning of the hyoid bone and tongue primarily contribute to these improvements. In another study on Twin Block therapy in children, a mean increase in upper airway dimensions was observed, from 14.16 mm to 15.6 mm post-treatment, particularly in the upper oropharynx. Both findings are consistent and support the airway-enhancing potential of functional appliances during growth [7, 8].

Fixed Functional Appliance

A fixed functional appliance (FFA) is effective in treating Class II malocclusion in non-compliant patients when performed during the SMI5, SMI6, and SMI7 stages of handwrist radiograph, where the maximum pubertal growth spurt occurs [9].

Evidence from a systematic review suggests that FFAs are effective in treating Class II malocclusion, especially when used during the pubertal growth phase. They work by promoting mandibular advancement and restraining maxillary growth. However, dentoalveolar changes, such as incisor inclination and molar movement, achieve most of the correction. Additionally, FFAs improve facial aesthetics by enhancing soft-tissue profiles such as the nasolabial and Holdaway angles [10].

The Herbst appliance uses a bilateral telescopic system to advance the mandible, generating distal forces on maxillary teeth and mesial forces on the mandible. It is one of the most widely used functional appliances for treating Class II malocclusions, proving effective even in difficult cases and with non-compliant patients when properly diagnosed and selected [11].

Complications associated with FFAs can be categorized into appliance-related and patient-related issues. Mechanical problems include breakage, spring fatigue, separation of components, and shearing of molar tubes. Patient-related effects involve soft tissue irritation, intrusion or rotation of teeth, and occlusal plane canting, which may impact treatment outcomes and comfort [12].

Extraction Protocol in Class II Management

Premolar extraction remains a well-established orthodontic modality for managing dental crowding, correcting malocclusion, and achieving favorable facial aesthetics. The severity of crowding, the patient’s facial profile requirements, and anchorage considerations influence the decision between first and second premolar extraction [13].

First premolar extraction allows for greater anterior retraction, provides superior anchorage control, and improves profile correction, especially in protrusive cases. However, potential drawbacks include excessive lip retrusion and possible reduction in airway dimensions. Conversely, extraction of the second premolars is associated with increased anchorage loss and limited retraction capacity, yet it preserves soft tissue fullness, making it more appropriate for cases with mild-to-moderate crowding [13].

Janson et al. (2014) demonstrated that both three and four-premolar extraction protocols result in comparable long-term occlusal stability in Class II subdivision malocclusions. However, the three-premolar approach provided superior efficiency, improved midline correction, and reduced patient compliance, making it a preferred option [14]. Mendes et al. (2019) reported that two-premolar extraction protocols yielded more favorable post-treatment profile attractiveness compared to four-premolar extractions [15].

Evidence suggests that orthodontic treatment with or without the extraction of premolars does not lead to problems with TMDs [16]. However, a cone-beam computed tomography (CBCT) study assessed three-dimensional (3D) condylar and joint space changes in Class II patients treated with maxillary first premolar extraction and incisor retraction. A significant posterior shift of the condyle and altered anterior and posterior joint spaces were observed, with no change in mediolateral or vertical position [17].

Management of Class III malocclusion

The Reverse Twin Block Appliance

The Reverse Twin Block appliance, often combined with lip pads and expansion, advances the maxilla and repositions the mandible posteriorly to correct Class III malocclusions. Compared with the facemask, it produces less dental compensation and more skeletal correction, though craniofacial changes are milder, especially in late mixed dentition, yet it remains effective with good patient compliance [18].

Class III Activator

Rakosi proposed a modified activator for managing Class III cases, made of wire and acrylic components. This appliance achieved significant skeletal correction that was maintained after treatment, with improvements in the skeletal profile remaining stable despite a compensatory decrease in the gonial angle and an increase in the maxillomandibular differential [19]. Short-term treatment effects included improvement of the patient profile with significant clockwise rotation of the mandible and proclination of the maxillary incisors [20].

Removable Mandibular Retractors

Researchers at the University of Florence, led by Tollaro and colleagues, were the first to assess the RMR appliance. This device has been proposed as a simple and functional treatment option for Class III malocclusions during both the primary and mixed dentition stages. Findings indicate that the RMR primarily produces dental rather than skeletal effects, and these changes can occur within a relatively short treatment period. However, the success of therapy is strongly influenced by patient cooperation, as consistent appliance use is essential for achieving optimal results [21].

Rapid Palatal Expansion and Facemask Therapy

Definitive orthodontic treatment is most commonly initiated during the late mixed or early permanent dentition stages to effectively correct malocclusions by utilizing the advantages of growth and leeway space [22].

Rapid maxillary expansion (RME) is a common treatment modality in correcting Class III malocclusion, aids in widening the maxillary bone, and triggers cellular reactions in the surrounding articulation. A successful approach to early treatment of Class III malocclusion involves the use of a protraction facemask along with a rapid palatal expander (RPE) appliance, especially in cases where insufficient maxillary growth is the main factor. This appliance induces forward-directed force to the maxilla, thereby indirectly promoting bone growth in the circummaxillary sutures, which remain open in younger patients [22].

Evidence from a systematic review suggests that facemask therapy with skeletal anchorage offers better maxillary advancement and stability compared to conventional facemask therapy. However, using tooth-borne RPE as anchorage has led to unfavorable outcomes, such as excessive inclination of the upper incisors and an increase in the lower third height. Introducing the Hybrid Hyrax bone-anchored RPE appliance offers a novel alternative treatment strategy to mitigate these complications [22].

Chin Cup Therapy

Early chin cup treatment promotes the limitation or redirection of posterior mandibular growth. Evidence suggests that chin cup therapy results in downward and backward movement of the mandible, along with overall development of the facial profile. According to the study, chin cup therapy improves the ANB and lower incisor to mandibular plane angle [23]. In a comparison between soft and hard chin cups, hard chin cups cause erythematous lesions of the skin [24].

Orthodontic Camouflage Versus Orthodontic‑Orthognathic Surgical Treatment

Camouflage treatment in orthodontics emerged in the 1930s-1940s, when extractions were commonly used to mask skeletal malocclusions. Growth modification was considered ineffective at that time, and surgical correction was still in its early stages [25]. The strategy to camouflage a Class III malocclusion usually involves proclination of the maxillary incisors and retroclination of the mandibular incisors to improve dental occlusion, although it might not correct the skeletal problem or facial profile [23].

Evidence based on the comparison of orthodontic camouflage versus orthognathic surgical correction suggests that orthognathic surgical treatment results in a clinically significant improvement in sagittal jaw relationship with a clockwise mandibular plane rotation, unlike the minimal effect seen with orthodontic camouflage. Additionally, camouflage treatment creates significant maxillary incisor proclination and mandibular incisor retroclination [26].

In adults, traditional orthodontic treatment for skeletal correction involves presurgical, surgical, and postsurgical phases, but this can temporarily worsen the facial profile due to preoperative decompensation. While surgical orthodontic treatment shows superior results in skeletal (ANB, SNB) and soft tissue profile improvement, orthodontic camouflage can be effective in mild skeletal corrections [25].

Orthognathic surgery

Orthognathic surgery is a widely used procedure in craniofacial surgery, including bilateral sagittal split osteotomy with or without osseous genioplasty and LeFort I osteotomy to address obstructive sleep apnea, malocclusion, and facial profile irregularities.

The surgery-first approach, pioneered by Nagasaka et al. in 2009, is increasingly being considered a viable treatment option suitable for patients with mild-to-moderate skeletal discrepancies and proper incisor alignment, offering shorter treatment times, improved outcomes, and stability. However, it can cause more mandible anticlockwise rotation, potentially affecting stability, making precise patient selection and presurgical planning essential [27].

Maxilla-first surgery produces a more precise 3D virtual treatment plan than mandibular-first surgery. Furthermore, both surgical methods display similar rates of relapse [28]. Evidence suggests that patients requiring bimaxillary osteotomies with a maxilla-first surgical approach tended to better align with the 3D virtual treatment plan compared to a mandibular-first operation [29].

Before 2003, the Craniofacial Centre predominantly employed the traditional two-splint orthognathic surgery method, utilizing two-dimensional surgical planning with plaster models and lateral and posteroanterior cephalograms. However, a shift occurred post-2003 toward adopting a one-splint approach. This innovative technique relies solely on the final occlusal splint for surgical guidance and involves the freehand positioning of the maxillomandibular complex [30]. Orthognathic surgery has experienced significant advancements owing to the incorporation of technology in preoperative preparation, particularly with the implementation of virtual surgical planning with shorter planning time, operating durations, and enhanced accuracy in osteotomies and fixation procedures [31]. Moreover, a significant correlation was noted between mandibular setback and the degree of relapse, suggesting that larger setbacks were associated with more noticeable relapse [31]. Conversely, no statistically significant correlation was observed with maxillary procedures. Likewise, there were no noteworthy distinctions in relapse between bimaxillary repositioning procedures and mandibular setback procedures performed individually [31].

Airway management in orthodontics

Functional appliance treatment in growing Class II patients has been shown to increase the anteroposterior measurements of the upper airway. A prolonged study including patients who underwent maxillary protraction, RME, and surgical correction reported a persistent enlargement of the nasopharyngeal airway [32].

In sleep apnea patients, mandibular advancement devices have been found to increase the pharyngeal airway and reduce the gap between the hyoid bone and the mandibular plane, resulting in improved Apnea-Hypopnea Index and reduced snoring [32].

Orthodontic therapy combined with extractions has little impact on an adult’s upper airway. There is no evidence to support the extraction of premolars in bimaxillary cases or crowding in adult patients, lowering the mean cross-sectional area of pharyngeal airway volume. The use of a TAD, miniscrew-assisted rapid palatal expander, specifically the tooth-borne RME or bone-borne RME designs, may result in large skeletal expansion and possibly major alterations to airway anatomy [33].

Rapid Maxillary Expansion and Airway in Children

RME improves nasal septum asymmetry and airway volume in children, while also reducing nasal airway resistance and enhancing airflow. However, it should be used alongside other treatments for conditions such as adenoid hypertrophy and septal deviation. Still, it shows no significant impact in adolescents with nasal septal deviation, suggesting that RME is more effective during childhood, which makes it a promising option for managing pediatric obstructive sleep apnea, as it effectively increases nasal cavity volume [34].

Temporary anchorage devices

TADs have become increasingly popular in orthodontic treatment to improve anchorage, expand the maxillary suture, and correct midline discrepancies. Miniscrews offer enhanced comfort and require less patient compliance compared to conventional anchorage devices. A major drawback of TADs is their unpredictable failure rate, ranging from 5% to 20%, which complicates identifying the exact factors causing their failure [35].

Recent advancements have introduced self-tapping screws and self-drilling screws. The self-tapping technique is preferred over the self-drilling approach for inserting miniscrews into the maxillary alveolar bone, with both methods demonstrating high success rates. Evidence suggests that self-tapping screws are more susceptible to fatigue than self-drilling screws because of differences in stress distribution [35]. Additionally, the use of 3D CBCT and stereolithographic methods for creating surgical guides is enhancing the precision of implant placement [36].

Utilizing skeletal anchorage to intrude and distalize the maxillary dentition, this treatment method offers a viable alternative for addressing high-angle skeletal Class II malocclusion and a gummy smile in adults, eliminating the necessity for orthognathic surgery [37].

Utilizing TAD for anterior en-masse retraction can lead to significant anchorage preservation compared to traditional methods. Evidence suggests that the skeletal anchorage device can achieve greater maxillary incisor intrusion compared to the Connecticut Intrusion Arch group [38]. Regarding molar distalization, there is no significant difference in duration between implant-supported and conventional tooth-supported distal jet appliances. Similarly, varying TAD locations, numbers, and appliance designs had no notable impact on molar distalization [39].

Does orthodontics cause temporomandibular disorders?

TMDs, a spectrum of clinical conditions involving pain or abnormal functioning of muscles used for chewing and the joints connecting the jaw to the skull, may result from abnormalities and discomfort in the temporomandibular joints (TMJs), masticatory muscles, and nearby tissues. The use of advanced 3D diagnostic imaging methods, including MRI, CBCT, and multidetector CT, is pivotal in understanding the development of TMJ dysfunction [40].

Orthodontic treatment has been suggested as a potential way to prevent or alleviate TMD by correcting jaw alignment and occlusal factors, though scientific evidence indicates that traditional orthodontic methods typically have a neutral effect on TMJ [41]. However, a systematic review by Alam et al. suggested that TMD patients had a higher chance of developing orthodontic problems than those without TMD [42]. Some researchers have suggested that functional mandibular advancement can positively impact TMJ reconstruction and improve the condyle-glenoid fossa relationship. Symptoms arising during functional appliance therapy typically resolve after treatment or during follow-up [40].

The evidence suggests that orthognathic surgery significantly reduces the incidence and severity of TMD, improving mouth opening without pain, though the type of jaw fixation may influence outcomes [43]. In contrast, Mehraban et al. (2020) reported no significant difference in TMD prevalence between patients who underwent orthognathic surgery and those who did not, though corrective treatments were linked to higher TMD occurrence compared to controls. They suggested that while dissatisfaction with appearance may contribute to TMDs preoperatively, improved aesthetics post-surgery could enhance self-esteem and psychological well-being, indirectly alleviating subjective TMD symptoms [44]. Treatment options include re-operation or condylectomy with reconstruction, while prevention focuses on patient education [44].

Deprogramming Appliances

Deprogramming is a process designed to alleviate jaw muscle tension and discomfort by reducing occlusal interferences. Findings revealed that individuals who received deprogramming splint therapy and occlusal equilibration experienced decreased clinical symptoms, accompanied by slight changes in condylar position [45].

TMD pain typically affects the masseter muscle, preauricular area, and anterior temporalis muscle, presenting as dull aches or sharp pain, especially during stress or teeth clenching. Treatment options include occlusal splints, physiotherapy, relaxation techniques, medications, arthroscopic surgery, and patient education. Physiotherapy may be particularly effective in reducing pain and improving the range of motion. Self-care practices, such as avoiding harmful oral habits and improving sleep, are vital for managing TMD symptoms. Stabilization splints can help relieve pain, but their effectiveness compared to placebos is still debated [46].

Pharmacological treatments such as cyclobenzaprine, non-steroidal anti-inflammatory drugs, and neuromodulatory drugs are used in complex cases, although their specific effectiveness for TMD remains uncertain. Psychological therapies and modalities such as acupuncture may provide short-term relief, but more research is needed [41].

Surgical treatments can be effective, similar to non-surgical options such as physical therapy, while the evidence for occlusal adjustment is lacking. Understanding TMD’s biopsychosocial aspects and prioritizing conservative management is crucial, with only a few cases needing invasive treatment. The International Classification of Orofacial Pain facilitates better risk prediction and treatment development based on TMD pathophysiology [41].

Maxillary expansion

RME, first described by Angell in 1860, was effective in inducing transverse skeletal changes, with the midpalatal suture opening accounting for 12-52.5% of the total screw expansion, leading to stable skeletal effects post-treatment. Long-term changes to the midpalatal suture after maxillary expansion are minimal, indicating the stability of the suture opening [47].

Compared to RME, slow maxillary expansion (SME) demonstrates superior efficacy in the molar region [47]. Unlike SME, RME causes anterior displacement of the maxilla and posterior rotation of the mandible. Neither technique causes root resorption or significant alterations in buccal and lingual bone thickness, although RME results in slightly greater posterior skeletal expansion [48].

Clear aligner therapy

Clear aligner therapy offers adults a discreet and comfortable alternative to braces, allowing easier oral hygiene and reduced plaque buildup. It lowers risks such as root resorption and early treatment pain while improving periodontal health. However, it may be less effective for severe or complex tooth movements [49].

Attachments in clear aligner therapy are crucial for enhancing aligner retention and facilitating tooth movement. Optimized rectangular horizontal attachments show promise for posterior anchorage, and bevelled-edge attachments aid in tooth movement and anchorage. For better outcomes, placing attachments on the lingual surfaces of anterior teeth and using high filler content composites with rigid transfer trays is recommended. Future research should focus on clinical tooth movement, new aligner protocols, and exploring hybrid treatment approaches [50].

Although clear aligners struggle with predictable control of rotation, inclination, and anterior extrusion, the introduction of Invisalign’s eighth-generation (G8) features (Align Technology, USA) represents a significant step toward improving these movements. Evidence suggests that using attachments across a broader range of teeth enhances posterior anchorage and derotation, with larger attachments yielding better results. Although clear aligners may not predict all tooth movements consistently, they can achieve clinically satisfactory results for minor adjustments, similar to fixed appliances [50].

The incidence of resorption ranged from 2% to 50%, with severe cases accounting for 22%. Clear aligner therapy may be associated with a lower chance of resorption than fixed therapy, particularly in cases where extractions are not needed. External root resorption in clear aligner treatments was significantly lower than in treatments with fixed appliances. Current evidence suggests that while clear aligners may not eliminate root resorption, both the incidence and severity of resorption could be reduced compared to results observed with fixed appliances [51].

Retention in orthodontics

Evidence from various studies offers insights into the efficacy and challenges of different types of orthodontic retainers. Fixed retainers are generally more effective than removable retainers after orthodontic treatment. Fixed retainers offer continuous support and stability, but with risks of failure. Removable retainers depend on patient compliance, which can be inconsistent. The strength of Hawley retainers does not significantly differ between part-time and full-time wearers [52]. Removable retainers are preferred for their lower failure rates and better periodontal health, but may cause discomfort. Computer-aided design/computer-aided manufacturing nitinol retainers provide better tooth stability, while fiber-reinforced composite retainers are more aesthetic but prone to earlier failure and increased gingival inflammation [53].

On evaluating the efficacy of lower arch bonded retainers in comparison to vacuum-formed retainers (VFRs), concerning treatment stability, initially, bonded retainers demonstrated superior performance compared to VFRs in the mandibular arch throughout the first six months of retention. Over the long term, bonded retainers exhibited a greater retention capacity than VFRs. In contrast, in the maxillary arch, both bonded retainers and VFRs demonstrated efficacy as retention methods for maintaining orthodontic treatment outcomes. Bonded retainers were significantly linked to increased plaque and calculus accumulation compared to VFRs in the short term, with both types exhibiting negative periodontal effects over the long run. Furthermore, both retainers exhibited similar failure rates in the maxillary arch throughout the initial year of retention. Nonetheless, VFRs demonstrated elevated failure rates in the upper arch compared to bonded retainers beyond this initial time. In contrast, the lower arch exhibited elevated failure rates for bonded retainers relative to VFRs [54].

Removable retainers are often recommended for part-time usage, approximately 9-10 hours daily. The proficiency of the operator is essential for the efficacy of fiber-reinforced composite retainers. Fixed retainers necessitate routine examinations for wire integrity and adhesion. The propensity for relapse with detachable retainers is approximately 40%. The failure rates for maxillary retainers average 41%, while those for lower retainers are 36%. Retainers must be affixed to all teeth using bonding agents, and regular evaluations are advisable, particularly during the initial six months [55].

Gaps in existing literature

Considering the widespread acceptance of evidence-based clinical practice in the field of orthodontics, it is apparent that there are significant limitations in the current literature. Newer treatment modalities such as clear aligners and TADs, along with digitalized treatment planning, lack adequate high-quality data [56]. Many current studies lack standardized techniques, have smaller sample sizes, and short follow-up periods, limiting their therapeutic applications. These gaps indicate the importance of comprehensive reviews to synthesize existing evidence and guide future, high-quality studies.

## Conclusions

Evidence-based practice in orthodontics is the application of the most recent and reliable evidence for making clinical decisions about patient care. The expanding field of scientific literature, marked by innovative research and findings, presents a challenge in staying updated on the latest developments. By consistently updating their knowledge and abilities, orthodontic professionals can ensure their practice is informed by the latest evidence, which increases the quality of treatment delivered to patients. The future of orthodontics depends on the integration of evidence-based decision-making, developing technology, and interdisciplinary collaboration. Utilizing evidence-based procedures guarantees superior treatment results and patient-centered care, while advancements such as digital imaging and 3D printing present promising opportunities for customized treatment strategies. The constant evolution of the orthodontic field necessitates the need for more high-quality evidence that will promote advancements and redefine the future of orthodontics.
